# Pin1 induces the ADP-induced migration of human dental pulp cells through P2Y1 stabilization

**DOI:** 10.18632/oncotarget.13377

**Published:** 2016-11-16

**Authors:** Soo-A Kim, Hong Seok Choi, Sang-Gun Ahn

**Affiliations:** ^1^ Department of Biochemistry, College of Oriental Medicine, Dongguk University, Gyeongju, South Korea; ^2^ College of Pharmacy, Chosun University, Gwangju, Republic of Korea; ^3^ Department of Pathology, School of Dentistry, Chosun University, Gwangju, Republic of Korea

**Keywords:** Pin1, P2Y1, MAPKs, human dental pulp cells, cell migration

## Abstract

PIN1, which belongs to a family of prolyl isomerases, specifically binds to phosphorylated Ser/Thr-pro motifs to catalytically regulate the post-phosphorylation conformation of its substrates. This study aimed to investigate the importance of Pin1 expression in human dental pulp cells (hDPCs) to understand the involvement of Pin1 in the regulation of P2Y1 and the activation of ADP-mediated P2Y1 signaling. This study found that the protein levels of P2Y1 gradually decreased after the onset of cell recovery following heat stress. Interestedly, hDPC migration significantly decreased during the recovery period. An *in vitro* study revealed that the silencing of PIN1 by siRNA or the pharmacologic inhibition of its activity decreased the migration of P2Y1 and P2Y1 expression in these cells. In addition, we found that Pin1 directly interacts with S252 of P2Y1 and that its binding stabilizes the P2Y1 protein to increase migration activity. These results strongly suggest that Pin1 mediates cell migration by stabilizing P2Y1 and that the Pin1/P2Y1 signaling pathways might serve as a novel mechanism of cell migration progression in hDPCs.

## INTRODUCTION

Human dental pulp cells (hDPCs) are multipotent cells with the potential to differentiate into a variety of cell types, including osteoblasts, chondrocytes, adipocytes, endothelial cells, neural progenitor cells, and myotubes [1, 2]. Recent studies have described the importance of hDPCs and their regenerative capacity in defective dental tissues [3, 4]. Pulpal regeneration in organ development and injured tissue involves the migration, proliferation and adhesion of hDPCs. Tissue-specific cytokines and other extracellular molecules can regulate cell regeneration [5, 6]. Various research groups have reported a role for adenosine nucleotide signaling through P2Rs in progenitor cell migration [7, 8]. For example, purinergic signaling modulates several biological functions in human bone marrow stem cells (hMSCs), including migration [7, 9, 10]. It has also been shown that human hematopoietic stem cells express several subtypes of functional P2XRs and P2YRs and that the stimulation of CD34+ cells by extracellular nucleotides improves their clonogenic capacity and migration in immunodeficient mice [11]. In particular, the migration of mouse glial progenitor cells has been shown to be regulated by the release of intracellular Ca^2+^, which is driven by ATP-induced P2Y1 activation [12]. P2Y1 receptors are G-protein coupled receptors that play a key role in intracellular Ca^2+^ hemostasis through phospholipase C (PLC)-mediated PIP2 hydrolysis and activation of the IP3 pathway [13]. In addition, the P2Y1 receptor is active in inflammatory responses and is associated with the migration of endothelial cells through activation of the small GTPase Rac1, reinforcing its relevance as an attractive target for new cardiovascular therapeutics [14, 15].

Pin1 is a peptidyl-prolyl cis/trans isomerase with an N-terminal WW domain that specifically binds to protein motifs containing proline residues preceded by a phosphorylated serine or threonine residue (pSer/pThr-Pro) [16]. Pin1 plays an important role in regulating cell proliferation, transformation, and ubiquitination and cell cycle progression, as well as the localization, stability, and interactions of subcellular protein and the phosphorylation status of targeted substrates [17]. Pin1 deregulation contributes to pathogenesis and human disease, particularly in cancers, including cervical, breast, liver, prostate, lung, and colon cancers [16]. Additionally, Pin1 plays pleiotropic roles in cardiac progenitor cells [18]. Pin1 has been shown to regulate various signaling pathways, including p53, β-catenin, NF-κB and Stat3, Cyclin D1, c-Jun, β-catenin, c-Myc, Raf kinase and APC signaling [19-22]. Pin1 also modulates the protein stability of estrogen receptor-alpha (ERα) in breast cancer, Nanog in embryonic stem cells and Oct4 in induced pluripotent stem cells [23]. However, very little information regarding its role in hDPCs is currently known.

The principal objectives of this study were to determine whether P2Y1 can stimulate hDPC migration and, if so, whether Pin1 is responsible for P2Y1-promoted cell migration. Second, we sought to characterize the mechanism through which Pin1 regulates P2Y1 receptor-mediated signaling. Our findings demonstrate that Pin1 stimulates the P2Y1-mediated migration of in hDPCs by stabilizing the P2Y1 protein and that Pin1 directly interacts with phosphorylated S252 in P2Y1, thereby regulating the ADP-stimulated migration activity of hDPCs.

## RESULTS

### Heat stress inhibits P2Y1 expression and cell migration

**Figure 1:**
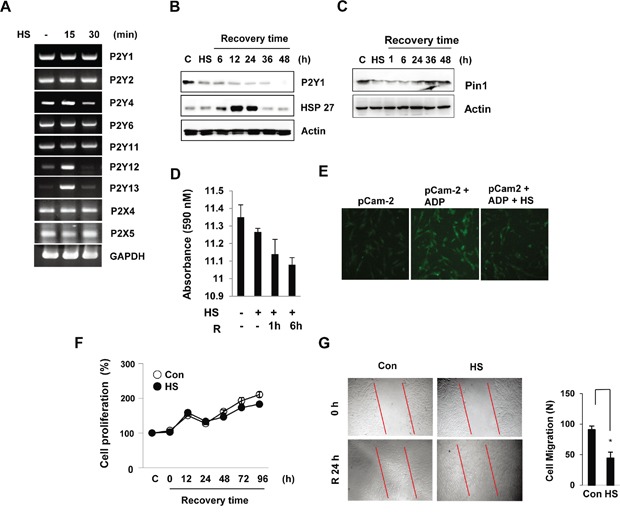
Thermal stress regulates P2Y1 expression in hDPCs hDPCs were subjected to thermal stress at 42°C for 15 or 30 min. **A.** The mRNA levels of the P2Y family genes were determined by RT-PCR using GAPDH as an internal control. **B, C.** Expression of P2Y1 and Pin1 during the recovery period after thermal stress. The cells were subjected to thermal stress at 42°C for 30 min, and the protein levels at various recovery time points were detected by Western blot analysis. The HSP27 levels served as a thermal stress control. **D.** Effect of thermal stress on P2Y1-mediated Ca^2+^ release. Culture media samples were collected, and the level of Ca^2+^ release was analyzed by ELISA. The bar charts show the quantification of Ca^2+^ secretion relative to the control. **E.** Measurement of Ca^2+^ signals using pCaMP2 plasmids in hDPCs. Representative confocal microscopy fluorescence images of HDPCs subjected to thermal stress (42°C) for 30 min. **F.** Effect of thermal stress on cell proliferation. Cell proliferation at the indicated recovery times after thermal stress (42°C for 30 min) was measured using an MTT assay. **G.** Wound-healing assays. Thermal stress inhibits wound healing in hDPCs. The images show representative results of three independent wound-healing experiments. The graphs show the quantitative evaluation of the migration rates. The data represent the means and S.D.s from three independent experiments. *P < 0.05 compared with the control.

A recent study reported that P2Y1 modulates the transduction of thermal stimuli through cutaneous polymodal nociceptors [25]. To determine whether thermal stress influences the expression of P2Y1 receptors in hDPCs, we performed RT-PCR using specific oligonucleotides against each of the P2Y1 family genes. After heat shock, no significant changes in the expression of P2Y1, P2Y2, P2X4, or P2X5 mRNA were detected. The levels of P2Y12 and P2Y13 were transiently up-regulated at 15 min in hDPCs (Figure 1A). The exposure of hDPCs to 42°C for 30 min induced few morphological signs of cellular toxicity. Therefore, a treatment protocol involving exposure to heat stress followed by a recovery period was used in the subsequent experiments. Importantly, a Western blot analysis revealed that the P2Y1 protein levels decreased after heat shock and consistently decreased during the recovery period. In contrast, the heat shock response marker Hsp27 was induced during the recovery time (Figure 1B). Interestingly, similarly to P2Y1 expression, the Pin1 levels in hDPCs also decreased after heat shock (Figure 1C). The ability of the P2Y1 receptor to mediate intracellular calcium mobilization in heat-treated hDPCs was investigated. The cells were exposed to 42°C for 30 min and allowed to recover at 37°C for 1 or 6 h. After the heat-shock recovery period, the hDPCs had reduced intracellular calcium levels (Figure 1D). In addition, we transiently transfected pCam2-EGFP plasmids that expressed GCaMP2 under the control of a CMV promoter and monitored the calcium release in hDPCs upon exposure to ADP based on EGFP expression. Significantly lower fluorescence was observed in the cells exposed to heat stress compared with the ADP-treated cells (Figure 1E).

To evaluate whether heat stress inhibits hDPC proliferation, cell proliferation was investigated using the MTT assay. The cells were subjected to heat shock (42°C) for 30 min and subsequently allowed to recover at 37°C, and the assay revealed that cell proliferation was not significantly altered during the recovery period. However, extension of the recovery period after heat stress to 24 h significantly decreased cell migration (Figure 1F and 1G).

### Effect of ADP on hDPCs migration

**Figure 2:**
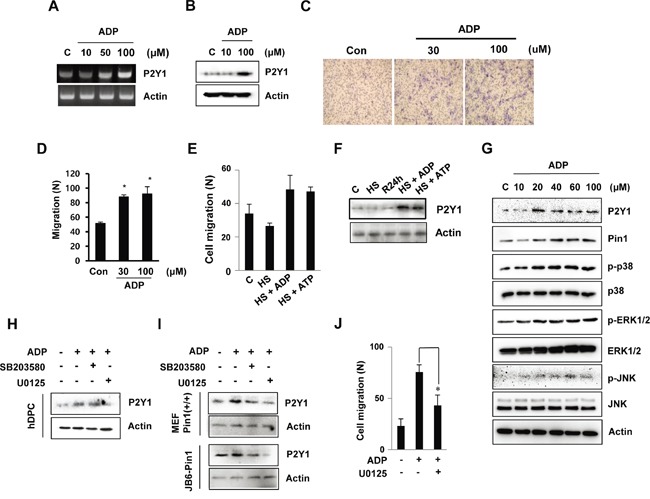
ADP-induced hDPC migration is involved in the MAPK pathway **A, B.** The mRNA and protein expression of the ADP-induced P2Y1 receptor in hDPCs was determined by RT-PCR and Western blot analyses. The cells were treated with ADP at the indicated concentrations for 24 h. **C, D.** *In vitro* migration assays. The cells were treated with ADP (30 or 100 μM) for 24 h, and the migration assays were performed in 12-well plates. After 24 h, hDPCs were fixed, stained with Mayer's hematoxylin, mounted, imaged and counted at 100X magnification using an optical microscope to assess the extent of migration. *P < 0.05 was considered significantly different from the control. **E, F.** Effects of ADP and ATP on the migration of and P2Y1 expression in cells subjected to thermal stress. The cells were exposed to 42°C for 30 min and incubated with or without 100 μM ADP or ATP for 24 h. The graphs show the quantitative evaluation of the migration rates. The levels of P2Y1 protein were detected by Western blot analysis. **G.** ADP stimulation of MAPK. The ERK1/2, JNK, and p38 phosphorylation levels in hDPCs stimulated with ADP at the indicated concentrations for 24 h were determined through Western blot analysis. **H, I.** Effects of MAPK inhibitors on ADP stimulation of MAPK pathways and cell migration. The phosphorylation levels of ERK1/2 and p38 in hDPCs stimulated with ADP (100 μM) for 24 h in the absence (control) or presence of 20 μM U0126 or SB203580 (SB) were determined by Western blot analysis. **J.** Effects of U0126 (20 μM) on ADP-stimulated hDPC migration. *P < 0.05.

To determine whether ADP induces P2Y1 expression and cell migration in hDPCs, we stimulated cultured hDPCs in a transwell migration chamber system. First, P2Y1 mRNA and protein levels were increased by ADP in a concentration-dependent manner (Figure 2A and 2B). By counting the number of cells that migrated through the membrane, we confirmed that ADP induces hDPC migration (Figure 2C and 2D). We further tested whether the migratory effect of ADP was mediated through P2Y1. Figure 2E shows that both ADP and ATP stimulated a heat-mediated decrease in cell migration. Similarly, both ADP and ATP induced a heat-mediated decrease in P2Y1 levels. These results indicate that ADP induces P2Y1 as a stimulatory factor for hDPC migration.

To identify the potential cell signaling pathways that are relevant to ADP-stimulated hDPC migration, we stimulated the cells with ADP and noted that ADP increased the phosphorylation levels of p38 and JNK in a concentration-dependent manner. Specifically, significant phosphorylation of both extracellular signal-regulated protein kinase (ERK)1/2 and p38 was observed after induction with 20 μM ADP, and maximal phosphorylation was observed with 100 μM ADP. ADP also increased the level of Pin1 with similar potency (Figure 2G). To investigate the role of MAPK in P2Y1 expression, hDPCs were pre-treated with the MAPK inhibitors U0126 and SB203580 prior to stimulation with ADP. As shown in Figure 2H, the ADP induction of P2Y1 was inhibited by U0126 but not by SB203580. In addition, U0126 lowered the P2Y1 levels in the Pin1-stable cell lines MEF Pin1^+/+^ and JB6-Pin1 (Figure 2I). The results show that ADP-induced P2Y1 expression is strongly correlated with ERK1/2 activation in cells.

We also found that ADP-stimulated cell migration was inhibited by U0126 (Figure 2J). However, suppression of the p38 pathway by SB203580 did not inhibit ADP-stimulated cell migration (data not shown). These results suggest distinct roles for the ERK1/2 pathways in ADP-promoted hDPC migration.

### Pin1 depletion inhibits P2Y1 protein expression and MAPK activity

**Figure 3:**
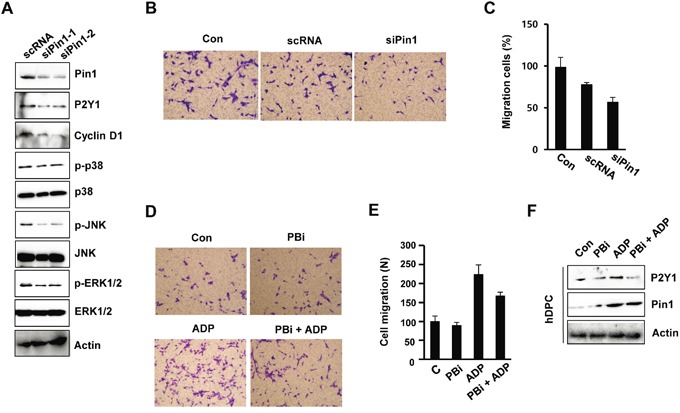
Pin1 depletion inhibits MAPK phosphorylation and cell migration **A.** Effect of siPin1 on p38, JNK, and ERK1/2 phosphorylation. hDPCs were transfected with Pin1-specific siRNA for 48 h, and the protein levels of p-p38, p-JNK, p-ERK1/2, and Pin1 were assessed. **B, C.** Effect of siPin1 on cell migration. The cells were transfected with siPin1 for 48 h. Migration assays were performed as described in the Materials and Methods section. The results represent the averages from three independent experiments. **D, E.** Effect of the Pin1 inhibitor PBi on ADP-induced migration. hDPCs were stimulated with ADP (100 μM) for 24 h in the absence or presence of 10 μM PBi. The graphs show the quantitative evaluation of the migration rates. **F.** hDPCs were treated with ADP and/or PBi for 24 h, and the protein levels of P2Y1 and Pin1 were assessed by Western blot analysis.

To examine the role of Pin1 in ADP/P2Y1-induced cellular responses, we knocked down endogenous Pin1 in hDPCs by siRNA. The knockdown was confirmed by immunoblotting and inhibited the phosphorylation of p38, ERK1/2, and JNK and the expression of P2Y1 (Figure 3A). These results indicate that Pin1 is involved in the principal events of the P2Y1 and MAPK signaling pathway.

To examine whether Pin1 is required for cell migration, the cells were transfected with Pin1-targeting siRNAs or treated with the Pin1 inhibitor PBi. The motility of the cells in which Pin1 was knocked down was delayed relative to that of the control cells, as determined through chamber migration assays (Figure 3B and 3C). We confirmed the effects of Olig2 knockdown using another siRNA duplex that targets Pin1 (data not shown). Similar results were observed in the Pin1 inhibitor (PBi)-treated cells. ADP-induced cell motility was affected by the inhibition of Pin1 activity in hDPCs (Figure 3D and 3E). A Western blot analysis showed that PBi inhibited ADP-induced P2Y1 expression, indicating that the machinery for ADP/P2Y1-mediated cell migration is affected by Pin1 activity (Figure 3F).

### Pin1 inhibits the heat shock-dependent degradation of P2Y1 proteins

**Figure 4:**
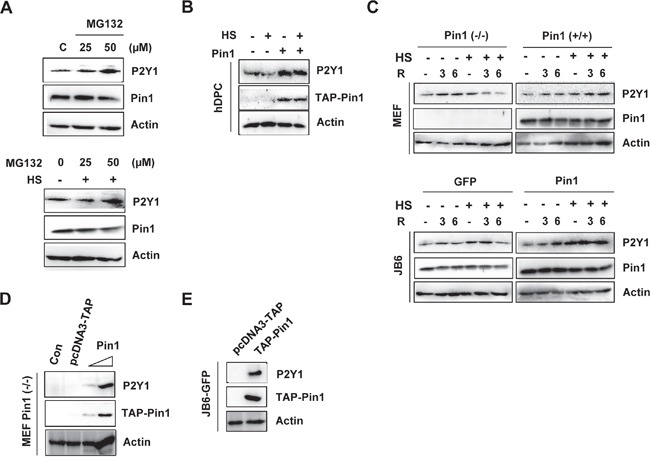
Loss of Pin1 accelerates proteasome-mediated degradation of P2Y1 **A.** hDPCs were treated with 25 or 50 μM MG132 with or without thermal stress for 24 h, and a Western blot analysis for P2Y1 and Pin1 was performed. **B.** The cells were transfected with TAP-Pin1 for 24 h, subjected to thermal stress and and incubated for another 24 h. A Western blot analysis was performed to assess the P2Y1 levels. **C.** Effect of Pin1 on heat-treated cell lines stably expressing Pin1. MEF WT, MEF Pin1^-/-^, JB6-GFP, and JB6-Pin1 cells were subjected to thermal stress for 30 min, and the P2Y1 and Pin1 protein levels at the indicated recovery time points were detected by Western blot analysis. **D, E.** Pin1 stabilizes P2Y1. MEF Pin1^-/-^ and JB6-GFP cells were transfected with 0.1 or 1 μg TAP-Pin1 plasmids for 48 h, and a Western blot analysis was performed to assess the P2Y1 protein levels.

Previous research has suggested a link between heat stress and the degradation of cellular proteins by the proteasome [26]. To investigate whether proteasome-dependent protein degradation is involved in the heat shock-mediated up-regulation of P2Y1 protein, we treated cells with the proteasome inhibitor MG132 or the vehicle control, DMSO. As shown in Figure 4A, the P2Y1 protein levels in MG132-treated cells were substantially higher than those observed in control cells. Our above-discussed data indicate that heat shock decreases the P2Y1 protein level. However, MG132 inhibited the heat shock-mediated reduction in the P2Y1 protein level. Because Pin1 stabilizes several cellular proteins, we questioned whether Pin1 is involved in the regulation of P2Y1 protein stability. To test this hypothesis, we transiently expressed Pin1 in hDPCs, and immunoblotting results showed that Pin1 expression led to increased P2Y1 protein levels, even after heat shock (Figure 4B).

We also examined whether Pin1 mediates the stabilization of P2Y1 in heat shock-treated MEF Pin1^-/-^ and Pin1^+/+^ cells and in GFP- and Pin1-overexpressing JB6 Cl41 (GFP-JB6 and Pin1-JB6) cells. As shown in Figure 4C, the presence of Pin1 completely restored the heat shock-mediated decline of P2Y1. The combination of the proteasome inhibitor MG132 and Pin1 stabilized the P2Y1 protein levels (data not shown).

In addition, we found that the transient expression of Pin1 in MEF Pin1^-/-^ and JB6 cells that were stably overexpressing GFP (JB6-GFP) up-regulated the protein level of P2Y1 (Figure 4D and 4E). However, the transfection control vector was not affected by the protein levels of P2Y1. These data suggest that Pin1 inhibits the heat stress-mediated degradation of P2Y1 proteins.

### Pin1 is critical for P2Y1 protein stabilization

**Figure 5:**
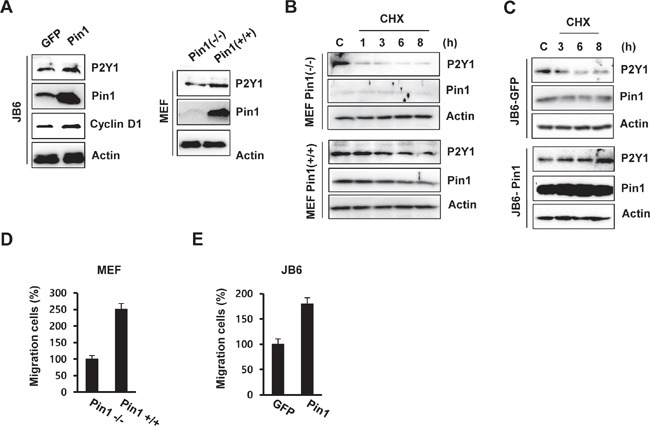
Pin1 up-regulates the level of P2Y1 protein by increasing its half-life **A.** Protein levels of P2Y1 and Pin1 in MEF Pin1^-/-^, MEF Pin1^+/+^, JB6-GFP, and JB6-Pin1 cells. **B, C.** Half-life of P2Y1 in the presence of Pin1. Immunoblot analyses were performed using whole-cell extracts of MEF and JB6 cells treated with or without CHX at the indicated times. **D, E.** Migration of MEF Pin1^-/-^, MEF Pin1^+/+^, JB6-GFP, and JB6-Pin1 cells. The migration assays are described in the Materials and Methods section. The results represent the averages from three independent experiments.

To further confirm the regulatory role of Pin1 in P2Y1 protein stabilization, an immunoblotting analysis was performed in Pin1^-/-^ and Pin1^+/+^ MEF cells and in GFP- and Pin1-overexpressing JB6 Cl41 (GFP-JB6 and Pin1-JB6) cells. The P2Y1 protein level was up-regulated in the Pin1-overexpressing MEFs and/or JB6 C141 cells compared with the level observed in Pin1^-/-^ MEF and JB6-GFP cells (Figure 5A).

To examine whether Pin1 affects P2Y1 protein stability, we measured the half-life of the P2Y1 protein in MEF and JB6 C141 cells through cycloheximide (CHX) treatment. As shown in Figure 5B, Pin1 extended the half-life of the P2Y1 protein in MEF Pin1^+/+^ cells. In contrast, P2Y1 stability was not affected in MEF Pin1^-/-^ cells under similar conditions. Data from subsequent experiments with stable JB6 C141 cell lines expressing wild-type Pin1 showed that P2Y1 has a longer half-life in JB6-Pin1 cells than in JB6-GFP cells (Figure 5C). Taken together, these data suggest that Pin1 stabilizes the P2Y1 protein.

We then examined whether Pin1 affects cell migration in MEF cells. As demonstrated through chamber migration assays, Pin1 knockdown abrogated cell migration (Figure 5D), and cell migration was induced in the Pin1-overexpressing JB6 C141 (JB6-Pin1) cells compared with the JB6-GFP cells (Figure 5E). These results are consistent with our observations of the ADP-regulated effect of Pin1 knockdown on P2Y1 expression.

### Pin1 interacts with P2Y1 protein

**Figure 6:**
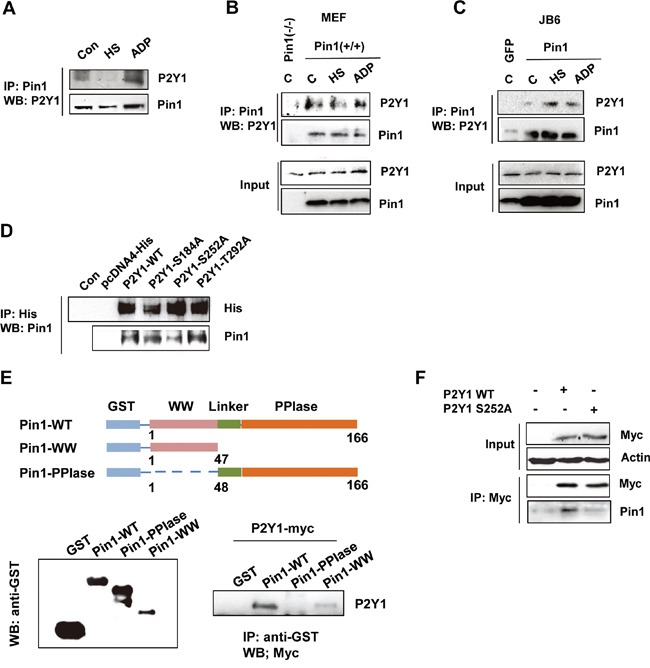
P2Y1 proteins physically interact with Pin1 **A.** Endogenous Pin1 interacts with P2Y1 in hDPCs. hDPCs were subjected or not subjected to thermal stress or treated or not treated with ADP for 24 h. hDPCs were lysed and used for immunoprecipitation (IP) with anti-Pin1 antibody. Pin1 or P2Y1 was visualized by immunoblotting. **B, C.** Pin1 strongly interacts with P2Y1 in MEF Pin1^+/+^ and JB6-Pin1 cells. Pull-down experiments were performed as described above. A total of 100 μg of total protein from the lysate pull-down products (top panel) and inputs (bottom panel) were subjected to IB analysis with P2Y1 and Pin1 antibodies. **D.** The S252 residue of P2Y1 mediates the Pin1–P2Y1 interaction. Three residues (S184, S252, and T292) were mutated to alanine. H1299 cells were transfected with either P2Y1 wild-type or mutant expression plasmids. The cells were lysed and subjected to a pull-down assay. Immunoblotting analysis was performed using an antibody specific for His and Pin1. **E.** P2Y1 binds to the WW domain but not the PPIase domain of Pin1. Schematic representation of full-length Pin1 and various truncation mutants. Whole-cell lysates derived from HEK293 cells expressing myc-tagged P2Y1 were incubated with glutathione-Sepharose beads containing GST, GST-Pin1-WT, GST-Pin1-WW, or GST-Pin1-PPIase. After washing, the bound proteins were subjected to immunoblot analysis with anti-myc antibody. **F.** hDPCs were transfected with either P2Y1 wild-type or P2Y1 S252 plasmids. The cells were lysed and subjected to a pull-down assay. Immunoblotting analysis was performed using antibodies specific for myc and Pin1.

Pin1 physically interacts with various phosphorylated proteins [19-22]. To test whether it can bind to the P2Y1 protein, we performed a Pin1 pull-down experiment, and the results showed that P2Y1 was clearly pulled down by Pin1 in ADP-treated hDPCs but not in heat-treated cells (Figure 6A). In addition, MEF and JB6 C141 cells stably expressing wild-type Pin1 and/or MEF Pin1^-/-^ cells were treated with ADP or subjected to heat stress to confirm the interaction between Pin1 and P2Y1. The cell lysates were immunoprecipitated using an anti-Pin1 antibody and blotted with an anti-P2Y1 antibody. As shown in Figure 6B and 6C, these two cell lines stably expressing Pin1 present comparable levels of exogenous wild-type Pin1. Immunoprecipitation assays revealed that endogenously expressed Pin1 bound to P2Y1 in cells stably overexpressing Pin1 but not in cells in which Pin1 was knocked down or in cells expressing low levels of Pin1. As mentioned above, Pin1 recognizes pSer/pThr-Pro motifs. An analysis of the P2Y1 protein sequence revealed three tentative pSer/pThr-Pro motifs (S184, S252, and T292) that might be recognized by Pin1. We created S184A-, S252A-, and T292A-mutant P2Y1 constructs and transfected cells with either wild-type or mutant P2Y1 constructs. P2Y1 binding by Pin1 was analyzed using a His-tagged P2Y1 pull-down assay. As shown in Figure 6D, the interaction between Pin1 and P2Y1 was decreased in the P2Y1 S252 mutant compared with the wild-type P2Y1 or other pSer/pThr mutants.

To determine the region of Pin1 responsible for its interaction with P2Y1, we then performed GST pulldown assays with GST-Pin1-WT, GST-Pin1-WW, and GST-Pin1-PPIase recombinant proteins, and the results showed that P2Y1 binds to the WW domain of Pin1 (Figure 6E). Similarly, we identified the interaction between Pin1 and P2Y1 in hDPCs (Figure 6F). These findings demonstrate that P2Y1 Ser252 phosphorylation is necessary for the interaction between Pin1 and P2Y1.

## DISCUSSION

The mammalian P2Y proteins are G-protein-coupled receptors that promote ADP-induced platelet aggregation, inositol lipid signaling, and the regulation of extracellular ions, such as Ca^2+^ [27, 28]. Recent reports have indicated that purinergic signaling, which is sensitive to ATP and its analogs, can modulate biological functions in human MSCs that express functional P2R subtypes [7, 9, 10].

The present study provides the first demonstration that ADP, an agonist of P2Y1 activation, stimulates hDPC migration. We also show that ADP activates MAPK signaling pathways, including ERK, JNK, and p38. The relative contributions of these MAPK pathways to cell migration are variable and depend on the cell type and the applied stimulus [29]. In hDPCs, ERK contributes to ADP-stimulated hDPC migration. Furthermore, the present study demonstrates that the ADP-stimulated ERK1/2 pathway mediates the expression of the P2Y1 protein.

P2Y receptors are linked to a variety of signal transduction mechanisms, including the phosphoinositide 3-kinase/Akt (PI3K/Akt) pathway and the ERK pathway [13, 30]. ATP and UTP stimulate the ERK and p38 pathways via the P2Y receptor [31]. It has been reported that the P2Y1 receptor is phosphorylated in an agonist-dependent manner in human platelets and Madin-Darby canine kidney (MDCK) epithelial cells. The effect of ATP on cell activation is blocked by PLC, PKC and ERK inhibitors, such as U73122, chelerythrine chloride, and PD98059, respectively [32-34]. Although some lines of evidence indicates that the post-translational regulation of P2Y1 is important for controlling P2Y1 activity, the mechanism through which the P2Y1 protein is regulated remains unclear. We hypothesized that in hDPCs, Pin1 supports cell migration through ADP-activated P2Y1 receptor cell signaling pathways.

We discovered that Pin1 is required for the proper migration of hDPCs. Through *in vitro* migration and Matrigel invasion assays, we found that cell migration was significantly inhibited by siPin1 and the Pin1 inhibitor PBi. Furthermore, Pin1 overexpression can prompt hDPC migration. It was recently reported that Pin1 is involved in odontogenic differentiation and adult tooth development of hDPCs through activation of BMP/Wnt/b-catenin/ERK signaling [35]. In conclusion, Pin1/P2Y1 might play a role in the differentiation, development, and migration of hDPCs through the ERK1/2 pathway.

The mechanism through which Pin1 regulates hDPC migration is currently unknown, and we identified a mechanism through which Pin1 affects P2Y1 activation and expression. The protein stability of P2Y1 is decreased by Pin1 knockdown, as was clearly observed with ADP-activated P2Y1. A possible explanation for these effects is that Pin1 specifically catalyzes the Ser/Thr-Pro phosphorylation sites of P2Y1 proteins and affects their activity, protein–protein interactions, or stability. Our results can be explained through two potential mechanisms. The first is that Pin1 facilitates the interaction of a kinase with P2Y1, thereby contributing to its phospho-activation or protein stability. Protein kinase C (PKC), which has been shown to phosphorylate P2Y1 at Thr339 in platelets and 1321N1 in human astrocytoma cells, is one candidate [32, 36]. Conversely, Pin1 might exert its effect by blocking the access of one or more phosphatases to the phosphorylated motif, resulting in these residues being more highly phosphorylated in the presence of Pin1. The kinase(s) and phosphatase(s) responsible for P2Y1 activation are currently unknown, and further studies are needed to identify the relevant receptor-regulated protein kinase or phosphatase responsible for P2Y1 activation.

As mentioned above, Pin1 is an important regulator of P2Y1. We found that Pin1 directly binds to P2Y1, resulting in P2Y1 protein stabilization. In addition, our results show that Pin1 enhances P2Y1-mediated cell migration. We examined whether the Ser/Thr-Pro motifs on P2Y1 are essential for its interaction with Pin1 and demonstrate that ADP-activated P2Y1 exhibits increased binding to Pin1 in hDPCs. We obtained similar results from a Pin1 pull-down assay using cell lines stably expressing Pin1. We also hypothesized that the ADP-induced phosphorylation of a residue(s) in P2Y1 plays a key role in P2Y1-promoted migration. Pin1-binding sites, specifically pSer/pThr-Pro motifs, were also identified in the P2Y1 protein. To identify which site is critical for Pin1 interaction, several P2Y1 point mutations, including S184A, S252A, and T292A, were constructed. As shown in Figure 6, wild-type P2Y1, S184A, and T292A all interacted with Pin1, whereas S252A P2Y1 exhibited a weaker interaction with Pin1. Our results identify S252 in P2Y1 receptors as a critical residue for Pin1 interaction in hDPCs. Although our data suggest that S252 is associated with Pin1 interaction, the phosphorylation state of S252 P2Y1 remains unclear. In future studies, we will examine the physiological significance of Pin1 interaction and S252 phosphorylation for P2Y1-mediated hDPC migration.

Additionally, to examine the expression of the P2X gene family in ATP- or ADP-treated hDPCs, we performed RT-PCR using specific oligonucleotides against each of the P2X family genes (P2X1-7). Four out of seven P2X genes were silenced in the control cells and in the ATP- and ADP-treated hDPCs. The mRNA expression levels of P2X4, P2X5 and P2X7 were higher in the ATP- and the ADP-treated cells compared with the control. Interestingly, the expression of P2X5 and P2X7 was higher in ATP-treated cells than in ADP-treated cells (Supplementary Materials). Therefore, our study shows that at least two P2X receptor isoforms are expressed by ATP in hDPCs and that these could play important roles in hDPC behavior via the purinergic receptor system.

Overall, this study explored the relevance of Pin1 on P2Y1-mediated cell migration. P2Y1 receptors and ERK activation mediate the effect of the P2Y1 agonist on hDPC migration. The interaction between Pin1 and P2Y1 enhances protein stability and increases the migratory capacity of hDPCs. We identified the S252 residue of P2Y1 as a strong candidate for Pin1 interaction. Thus, Pin1/P2Y1 might be a novel important modulator of hDPC migration that could prove useful in dental tissue regeneration.

## MATERIALS AND METHODS

### Cell culture and reagents

hDPCs were kindly provided by Prof. Takashi Takata (Hiroshima University, Japan) and maintained in DMEM (Invitrogen) supplemented with 10% fetal bovine serum (FBS), 100 U/ml penicillin and 100 μg/ml streptomycin, as described previously [1]. Pin1^+/+^ and Pin1^−/−^ MEF cells were kindly provided by Dr. Kun Ping Lu (Beth Israel Deaconess Medical Center, Harvard Medical School) and maintained in DMEM with 10% FBS. JB6-GFP and JB6-Pin1 mouse epidermal cells were cultured in Minimal Essential Medium (MEM, Sigma-Aldrich, St. Louis, MO, USA) supplemented with 5% FBS. The cells were maintained in DMEM or MEM supplemented with 10% FBS (Biochrom AG, Berlin, Germany), 100 U/ml penicillin and 100 μg/ml streptomycin (Sigma) at a temperature of 37°C with 5% CO_2_. SB203680, U0125 and PBi were purchased from Sigma-Aldrich. The Fugene HD transfection reagent was obtained from Promega (Madison, WI, USA). MG123 was purchased from Calbiochem. Polyclonal anti-Pin1 and P2Y1 antibodies were obtained from Santa Cruz Biotechnology, Inc. (Dallas, TX, USA). Monoclonal anti-phospho-ERK1/2, anti-phospho-p38, anti-phospho-JNK, anti-ERK1/2, anti-p38, and anti-JNK antibodies were purchased from Cell Signaling Technology (Danvers, MA, USA). The monoclonal myc antibody was obtained from Santa Cruz Biotechnology, Inc. Mutant P2Y1-S182, S252, and T292 plasmids were constructed using the QuikChange^®^ site-directed mutagenesis kit (Agilent, Santa Clara, CA, USA).

### Construction of a mutant P2Y1 receptor and Pin1 siRNA

The full-length human P2Y1 receptor was cloned into a pcDNA4 expression vector with a C-terminal myc/His tag. The forward primer was 5’-ggggaattcATGACCGAGGTGCTGTGGCC-3', and the reverse primer without a stop codon was 5'-gcctcgagGCACAGGCTTGTATCTCCATTCTG-3'. The sense and antisense primers contained EcoRI and XhoI restriction sites, respectively, at their 3′ ends. The P2Y1-S182, S252, and T292 mutants were constructed using 5′ and 3′ primers up to 30 bases in length in which the indicated Ser and Thr codons were mutated to Ala. The constructs were confirmed by DNA sequencing. The full-length human Pin1 cDNA was cloned into a pcDNA3-TAP vector as described previously (24). Small interfering RNAs (siRNAs) were synthesized by GenePharma (Shanghai, China). The target sequence for siPin1 was GCCATTTGAAGACGCCTCG. The specific siRNAs or scrambled controls were transfected with Lipofectamine 2000 iRNA/Max Transfection Reagent (Invitrogen, Carlsbad, CA, USA) according to the manufacturer's instructions. The cells were harvested 24 h after transfection. Total cell lysates were separated by SDS–PAGE and analyzed by Western blot, as described below.

### Reverse transcription-polymerase chain reaction

hDPCs were subjected to heat shock (42°C) for 15 or 30 min. The total RNA of the hDPCs was extracted using the Trizol reagent (Life Technologies, Gaithersburg, MD, USA) according to the manufacturer's instructions. A total of 1 μg of total RNA was reverse-transcribed to cDNA in a reaction mixture of 20 μl with the Reverse Transcription System (Promega, Leiden, The Netherlands). Amplification was performed for 30 cycles in a DNA thermal cycler. GAPDH was used as a control for the PCR reaction. The primer sequences for P2Ys or P2Xs are detailed in Table 1. The PCR products were resolved on a 1.5% agarose gel and stained with ethidium bromide. The gels were scanned, and the bands were quantified by optical densitometry.

**Table 1:** Primers list of P2X receptors

Gene	Primers (forward and reverse)	Accession number in GenBank	Product size (bp)
P2X1	5′-CCAGCTTGGCTACGTGGTGCAAGA-3′ 5′-ACGGTAGTTGGTCCCGTTCTCCACAA-3′	U45448	226
P2X2	5′-CCCGAGAGCATAAGGGTCCACAAC-3′ 5′-AATTTGGGGCCATCGTACCCAGAA-3′	AF190823	208
P2X3	5′-CCCCTCTTCAACTTTGAGAAGGGA-3′ 5′-GTGAAGGAGTATTTGGGGATGCAC-3′	NM002559	245
P2X4	5′-CCTTCCCAACATCACCACTACTTACC-3′ 5′-AGGAGATACGTTGTGCTCAACGTC-3′	U85975	256
P2X5	5′-AGCACGTGAATTGCCTCTGCTTAC-3′ 5′-ATCAGACGTGGAGGTCACTTTGCTC-3′	AF016709	183
P2X6	5′-ATGGCCCTGTCCAAGTTCTGACAC-3′ 5′-TGTTGCCTCATCCTTGCTTTCGCT-3′	AF065385	140
P2X7	5′-CTGCTCTCTTGAACAGTGCCGAAA-3′ 5′-AGTGATGGAACCAACGGTCTAGGT-3′	Y09561	270
GAPDH	5′-GAAGGTGAAGGTCGGAGTC-3′ 5′-GAAGATG GTGATGGGATTTC-3′	NM002046	242

### Calcium measurements

Ca^2+^ measurements using GCaMP2-based constructs (pN1-G-Camp2 plasmids were provided by Dr. Choi, Chosun University) were performed through fluorescence microscopy with a CCD camera (IX-71, Olympus). Prior to the calcium measurements, the cells were seeded for 24 h onto a four-well Nunc Lab-Tek II Chambered Coverglass (Nalge Nunc International, Rochester, NY, USA). For the heat shock treatment, the cells were subjected to a temperature of 42°C for 30 min and allowed to recover at 37°C for 24 h in culture medium. The calcium ion (Ca^2+^) concentration was measured using a QuantiChrom™ calcium assay kit (DICA-500, BioAssay Systems, Hayward, CA, USA). For the analyses, 200 μl of working reagent was added to 5-μl aliquots of the sample or standard in a 96-well plate. The plate was incubated for 3 min at room temperature and then read at 595 nm using a microplate reader (Tecan Sunrise, Switzerland). Serial dilutions of CaCl_2_ (0–200 μg ml^−1^) were used to create a standard curve. The calcium loss was calculated in reference to the Ca^2+^ concentration of the solutions incubated without sample.

### Cell proliferation assay

hDPC proliferation was assessed through a 3-(4,5-dimethylthiazolyl-2)-2,5-diphenyltetrazoliumbromide (MTT) assay. The cells were seeded in a 96-well plate at a density of 1 x 10^4^ cells/well. The next day, the cells were washed twice with PBS, and 500 μg/ml MTT (Sigma) was added to the wells. The MTT solution was removed after 4 h of incubation at 37°C. A mixture of 0.01 M glycine and DMSO (Sigma) was added to each well. The absorbance was measured at 540 nm with a Benchmark microplate reader (BioRad Laboratories).

### Immunoprecipitation

Total cell extracts were incubated with anti-Pin1 in NP-40 lysis buffer (0.5% NP-40, 0.5% Triton X-100, 150 mM NaCl, 50 mM Tris HCl pH 7.4, 1 mM EDTA, 50 mM NaF, 1 mM B-glycerophosphate, 1 mM sodium orthovanadate, 0.5 μg/ml leupeptin, 1 μg/ml pepstatin, and 0.2 mM PMSF). The extracts were incubated overnight at 4°C with rotation and then with 20 μl of protein A/G beads (Life Technologies) for 3 h at 4°C. The beads were washed three times with the same buffer and suspended in 2X SDS sample buffer. The samples were resolved in SDS-polyacrylamide gels for Western blot analysis with specific antibodies as indicated. For GST pulldown assays, the cells were transfected with the P2Y1 plasmid. Whole-cell lysates were prepared 48 h after transfection and incubated at 4°C for 4 h with glutathione-Sepharose beads (GE Healthcare) containing GST-Pin1 and GST. The precipitated proteins were separated by SDS-PAGE, and protein expression was detected by immunoblotting.

### Western blot analysis

Cell lysates (50 μg) were placed in lysis buffer (50 mM Tris-HCl, pH 7.5, 150 mM NaCl, 1% NP-40, 0.5% Na-deoxycholate, 0.1% SDS, and a protease inhibitor cocktail containing 1 μg/ml aprotinin and leupeptin) and separated by 12% SDS-PAGE. The resolved proteins were transferred onto a nitrocellulose membrane (Amersham Pharmacia Biotech, UK) according to standard procedures. The membrane was blocked in 5% non-fat dry milk for 3 h and incubated with anti-Pin1, anti-P2Y1, anti-phospho-ERK, anti-ERK, anti-phospho-p38, anti-p38, anti-phospho-JNK, or anti-JNK antibodies for 3 h at RT. After incubation with the specific peroxidase-coupled secondary antibody (Sigma-Aldrich) for 1 h, the blotted bands were detected using an enhanced chemiluminescence detection kit (Amersham Pharmacia Biotech).

### Wound-scratch and cell migration assays

The cells were allowed to grow in a culture dish overnight, and a scratch approximately 3 mm in width was created in the monolayer using a pipette tip. After washing twice with PBS, the cells were subjected or not subjected to heat shock (42°C for 30 min), and images were captured after 24 h. The migratory behavior of the hDPCs was assessed using a transwell migration assay (Chemotaxis Cell Migration Assay kit, Chemicon). hDPCs were seeded onto Thincert™ tissue culture inserts (pore size = 8 μm; Greiner Bio-One, Frickenhausen, Germany) in DMEM with 10% FBS at a density of 1.5 x 10^5^ cells per insert. The cells were subjected or not subjected to heat shock and allowed to migrate for 24 h. Under some conditions, inhibitors against Pin1 and MAPKs were also added to the hDPCs. After 24 h, the hDPCs that had transmigrated towards the lower surface of the filter were fixed with methanol, stained with hematoxylin for 5 min and washed with PBS. The cells in five random microscopic fields per well were imaged using an Olympus IX2-SLP inverted microscope (Japan) at 100X magnification. The numbers of migrated cells on the lower side of the membrane were counted in five randomly selected areas, and the values are expressed as the mean area percentage.

### Statistical analyses

All experiments were performed at least three times. The mean values from the experiments are expressed as the means ± SE. Significant differences were assessed through analyses of variance. *P* < 0.05 was considered significant.

## SUPPLEMENTARY MATERIALS FIGURE


