# Supplementary material for: Pin1 induces the ADP-induced migration of human dental pulp cells through P2Y1 stabilization
Source: Oncotarget. 2016 Nov 16;7(51):85381–92. doi: 10.18632/oncotarget.13377 (PMC5356743; doi:10.18632/oncotarget.13377)
Supplement: Supplementary file 1 [file oncotarget-07-85381-s001.pdf]

Pin1 induces the ADP-induced migration of human dental pulp cells through P2Y1 stabilization

SUPPLEMENTARY FIGURE

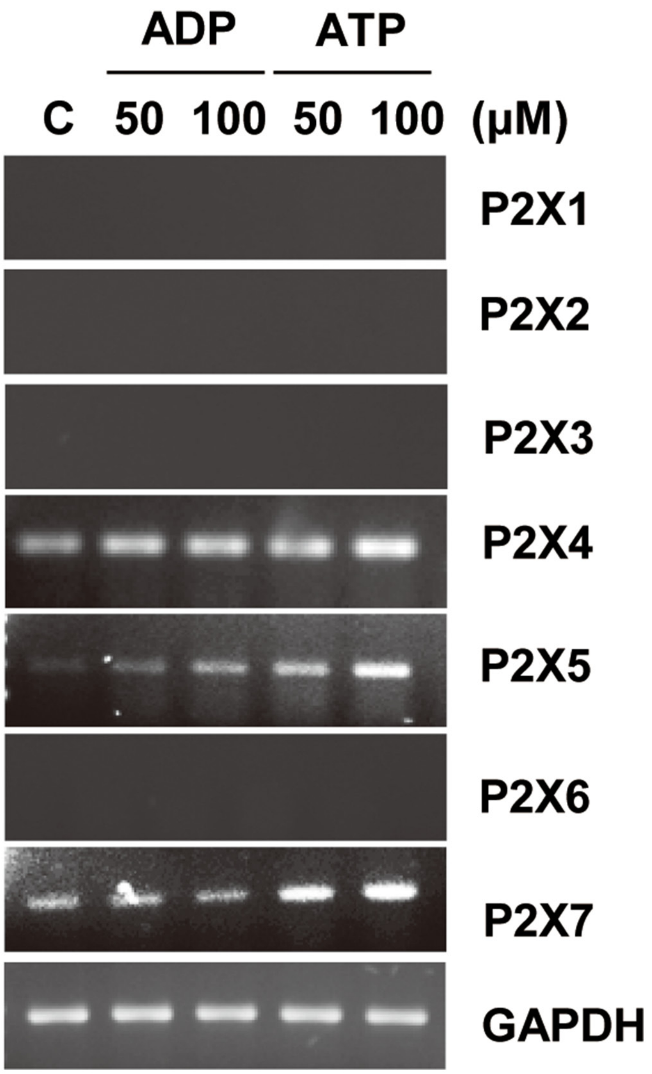

Supplementary Figure 1: Expression of P2Xs gene family in hDPCs. Cells were treated with ADP or ATP for 24 h. RT-PCR was performed using primers as described in Table1. GAPDH was used as an internal control.
